# Low Hepatic Mg^2+^ Content promotes Liver dysmetabolism: Implications for the Metabolic Syndrome

**DOI:** 10.4172/2167-0943.1000165

**Published:** 2014-10-01

**Authors:** Chesinta Voma, Zienab Etwebi, Danial Amir Soltani, Colleen Croniger, Andrea Romani

**Affiliations:** 1 Department of Physiology and Biophysics, Case Western Reserve University, USA; 2 Department of Clinical Chemistry, Cleveland State University, USA; 3 Department of Nutrition, Case Western Reserve University, USA

**Keywords:** Dietary magnesium, Hepatic Mg^2+^ homeostasis, Metabolic syndrome, Glucose 6 phosphate, Glucose 6 phosphate dehydrogenase, 11 Beta-hydroxysteroid-dehydrogenase-1, Cortisol

## Abstract

Metabolic Syndrome, a pathological condition affecting approximately 35% of the USA population, is characterized by obesity, insulin resistance, and hypertension. Metabolic syndrome is considered the single most common condition predisposing to the development of various chronic diseases including diabetes and hypertension. Hypomagnesaemia has been consistently observed in association with metabolic syndrome, but it is unclear whether reduced Mg^2+^ levels are the consequence or a possible cause for the development of the metabolic syndrome and/or its associated pathologies.

Research performed in our laboratory showed that rats exposed for 2 weeks to a Mg^2+^ deficient diet presented decreased glucose accumulation into the hepatocytes together with low Mg^2+^ level in the circulation and within the liver cells. To better investigate the changes in glucose metabolism, HepG2 were used to mimic in vitro Mg^2+^ deficiency conditions. HepG2 cells cultured in low extracellular Mg^2+^ presented a 20% decrease in total cellular Mg^2+^ content, reduced glucose accumulation, and enhanced glucose 6-phosphate (G6P) transport into the endoplasmic reticulum (ER). The increased G6P transport was associated with its enhanced hydrolysis by the glucose 6-phosphatase, but also conversion to 6-phosphogluconolactone by the glucose 6-phosphate dehydrogenase. The latter process resulted in the increased generation of NADPH within the ER and the increased conversion of cortisone to cortisol by the 11-β-hydroxysteroid dehydrogenase type-1 (11-β-OHSD1).

Taken together, our results provide compelling evidence that Mg^2+^ deficiency precedes and actually promotes some of the hepatic dysmetabolisms typical of the metabolic syndrome. The decrease in intrahepatic Mg^2+^ content up-regulates G6P entry into the hepatic endoplasmic reticulum and its routing into the pentose shunt pathway for energetic purposes. The associated increased in NADPH production within the ER then stimulates cortisol production, setting the conditions for hepatic insulin resistance and further altering liver metabolism.

## Introduction

In the last twenty years the incidence of obesity and type-2 diabetes has dramatically increased in both industrialized and developing countries. In the US, it is currently estimated that more than one-third of the US population is overweight or frankly obese [1], with an economic impact on health care costs estimated at ~$147 per year [2]. To this figure it has to be added the cost of severe medical complications associated with obesity, which include myocardial infarction, cardiovascular diseases, stroke, hypertension, and some forms of cancer (colon and breast cancer, preeminently).

Metabolic syndrome, also known as insulin resistance syndrome, or syndrome X, represents a particular form of obesity. First introduced by Dr. Haller in 1977 [3], the term refers to a cluster of disorders that includes dyslipidemia, hyper-lipoproteinemia, hepatic steatosis, hypertension, obesity, and insulin resistance (or glucose intolerance), and ultimately results in the onset of type 2 diabetes [4]. This definition reflects the view that insulin resistance is the underlying cause of the dysmetabolism [5], and leads to other complications including hypertension [4].

Because of the central role of insulin resistance in the pathophysiology of the metabolic syndrome, the liver becomes a vital organ in coordinating carbohydrate, lipid, and protein metabolism at the whole body level under tight control by circulating hormones such as insulin, catecholamine, cortisol, and glucagon [4]. Abnormal increase in intrahepatic lipid metabolites content directly inhibits insulin-stimulated glucose transport activity in the organ, with major implications for the onset of liver dysmetabolism in the metabolic syndrome.

Interestingly, the increase in the incidence of metabolic syndrome and obesity has coincided with the progressive decrease in dietary magnesium intake [6]. The current Western diet contains approximately 30% to 40% less magnesium than the diet in the late seventies [6] as a result of changes in food processing and water purification. Magnesium is the fourth most abundant cation in the human body, and the second within the cells after potassium [6]. Its distribution is such that approximately 99% of the total magnesium content is within bones, muscles, and soft tissues, leaving ~1% in the circulation [6,7]. Because of this distribution, serum magnesium level is not a reliable indicator of whole body magnesium homeostasis [6].

At the cellular level, magnesium ions (Mg^2+^) are highly compartmentalized within the cytoplasm, mitochondria, nucleus, and endoplasmic (or sarcoplasmic) reticulum [6,7]. Within these compartments Mg^2+^ is associated with phospholipids, chromatin, ATP, and other phosphonucleotides [7], where by total Mg^2+^ concentrations range between 15 and 18 mM within the cellular organelles, and between 4 to 5 mM in the cytoplasm [7].

Although elevated, these Mg^2+^ concentrations are not static but change dynamically following hormonal stimuli and metabolic conditions [7]. Administration of insulin, for example, promotes Mg^2+^ influx into the hepatocytes [7].

About twenty years ago, Resnick observed that Mg^2+^ content was decreased and Ca^2+^ content increased in erythrocytes from individuals affected by metabolic syndrome [8]. This prompted Resnick to propose the ‘ionic theory’ whereby these changes in Ca^2+^ and Mg^2+^concentrations were essential components of the metabolic syndrome. Although Resnick’s initial observation was confirmed by several other reports [9], it is still unclear whether Mg^2+^ deficiency proceeds or is a consequence of the metabolic syndrome onset.

In the present study, HepG2 cells were maintained in culture in the presence of physiological (1 mM) or reduced (0.6 mM) extracellular Mg^2+^ to mimic Mg^2+^ deficiency conditions. Changes in glucose accumulation and utilization were assessed by a combination of radioisotopic distribution techniques or fluorescence methods. The results reported here indicate that Mg^2+^ deficient hepatocyte present reduced glucose accumulation and enhanced G6P entry into the ER of the hepatocyte. This increased G6P entry is associated with an enhanced oxidation to 6-phosphogluconolactone via G6PD, and NADPH production. The production of NADPH, in turn, promotes a marked increase in the conversion of cortisone to cortisol via 11-β-hydroxysteroid-dehydrogenase-1. Taken together, these results suggest that a reduction in intrahepatic Mg^2+^ content changes glucose utilization within the hepatocyte and promotes cortisol production perhaps for gluconeogenetic purposes, setting the conditions for reduced insulin responsiveness.

## Materials and Methods

### Materials

HepG2 cells were a kind gift from Dr. Cederbaum (Mt. Sinai, New York). 3H-2-deoxyglucose was from Amersham (GE, Pittsburgh, PA). Culture medium and bovine calf serum were from Gibco (Life Science, Grand Island, NY). Antibody anti Glut2 and Glut1 glucose transporters were from Santa Cruz (Dallas, TX). All other reagents were of analytical grade (Sigma, St Louis, MO).

### Methods

#### Cell cultures

HepG2 cells were maintained in MEM medium (M2279, Sigma) containing 0.8mM MgSO_4_ and 5.5 mM glucose in the presence of 5% CO2 until 85% confluent. Cells were then divided into two groups and cultured in the presence of physiological (0.8 mM) extracellular Mg^2+^ or under Mg^2+^ deficient conditions (0.4 mM extracellular Mg^2+^). The presence of 10% bovine calf serum increased extracellular Mg^2+^ content to 1 mM and 0.6 mM, respectively (measured by atomic absorbance spectrophotometry).

At confluence, cells were harvested and used to determine Mg^2+^ and ATP content, glucose accumulation, or glucose 6 phosphate dehydrogenase activity.

##### Cellular Mg^2+^ content

To measure total cellular Mg^2+^ content, normal or Mg^2+^ deficient HepG2 cells were harvested at confluence, washed briefly in PBS (500 rpm for 1 min) and sedimented again (500 rpm for 2 min) in microfuge tubes. The supernatants were removed and the cell pellets dissolved in 10% HNO_3_. The acid mixture was sonicated for 10 min in a sonicating bath, and acid-digested overnight. The following morning, the acid mixtures were sonicated again, vortexed, and sedimented at 5000 rpm for 3 min in microfuge tubes. The supernatants were removed and assessed for Mg^2+^ content in a Varian 210 atomic absorbance spectrophotometer (AAS) calibrated with Mg^2+^ standards.

To measure the intracellular Mg^2+^ distribution, HepG2 cells were harvested and washed as described above. The cell pellets were then resuspended in a Mg^2+^ free medium containing (mM) 100 KCl, 10 mM Mops, 1mM KH2PO_4_, pH 7.2 [10], pre-warmed at 37°C, at the final concentration of 0.5 mg protein per ml. Digitonin (50μg/ml), FCCP (2 μg/ml), and A23187 (1μg/ml) were sequentially added to the cell mixture at 5 min intervals to measure cytoplasmic, mitochondrial, and post mitochondrial Mg^2+^ content, respectively [11]. Prior to the addition of any of these agents, aliquots of the cell mixture were withdrawn in duplicate and sedimented in microfuge tubes (5000 rpm for 3 min). The supernatants were removed and assessed for Mg^2+^ content by AAS. The pellets were digested in 10% HNO_3_ and assessed for Mg^2+^ content as reported above. Although indirect, this approach has been successfully used in our laboratory, with results equivalent to those reported by other with different techniques [11].

#### ATP content

Cellular ATP content was measured by luciferin-luciferase as previously reported [12]. Briefly, HepG2 cells were harvested, washed and sedimented as described above. The cell pellets were then extracted in 10% PCA on ice for 10 min. The mixture was neutralized with one volume of 1M KHCO_3_ on ice and sedimented in a refrigerated Beckman J6B centrifuge (1800 rpm for 5 min). The supernatant was removed and stored at −20°C until tested. The cellular ATP content was determined by luciferin-luciferase assay (detecting sensitivity in the pmol-nmol/ml range (Sigma) in a LUMAT Berthold LB 9,501 luminometer.

#### Glucose utilization

Glucose content in the culture medium was measured by glucose kit (Fisher) according to the directions of the producer. Alternatively, glucose accumulation into the cells was measured radio-isotopic distribution. Cells at confluence were serum starved for 3 hours prior to measure glucose accumulation. Cells were then harvested and washed as previously reported. The cell pellets were resuspended at the final concentration of 0.5mg protein/ml in a medium having a composition similar to the MEM medium reported above but containing only 100 μM glucose labeled with 0.5 μCi/ml3H-2-deoxyglucose, at 37°C [13]. At selected time points, 0.5 ml aliquots of the incubation mixture were withdrawn in duplicate and diluted 11 fold in 250 mM ‘ice-cold’ sucrose containing 20 μM phloretin as a glucose transporter inhibitor. The mixture was rapidly filtered onto Whatman glass fiber filters (GF, 250 nm pore size) under vacuum suction. The filter was washed once under vacuum with 5 ml of ‘ice-cold’ sucrose (plus phloretin), removed from the filtration device and air-dried. The radioactivity retained onto the filter was measured in a Beckman 6500 β-scintillation counter. For comparison purposes, similar experiments were carried out with glucose labeled with 3H-3-methyl-glucose or 14C-glucose.

#### Glucose transporter expression

The protein expression of Glut2 and Glut1 transporters was assessed by Western blot analysis using commercially available antibodies against these transporters (Santa Cruz). The blots were routinely stained with Ponceau-S before immune-staining to confirm samples loading and the transfer of equivalent amounts of protein. The membranes were extensively washed with PBS, and the primary antibody binding visualized using peroxidase-conjugated secondary antibody and enhanced chemiluminescent Western blotting detection reagents (Amersham). Densitometry was performed using Scion Image Program (NIH). The bands corresponding to Glut2 and Glut1 isoforms were separately ‘framed’ for densitometry determination. The obtained results were normalized for GAPDH gene product expression.

#### Glucose 6-Phosphate dehydrogenase activity

Glucose 6-phosphate dehydrogenase activity was measured as reported by Marcolongo et al. [14]. Briefly, HepG2 cells at confluence were harvested and washed as previously reported. The cells were then resuspended in a medium containing (mM): 100 KCl, 10 MOPS, 1 KH2PO_4_, pH 7.2, and permeabilized by the addition of 50μg/ml digitonin. Permeabilization was assessed by Trypan Blue exclusion test [12]. Aliquots of the cells were diluted 20-fold in a final volume of 2 ml medium, and transferred to a Perkin Elmer 3 fluorimeter (Perkin Elmer, 3) at 340nm excitation wavelength and 490 nm emission wavelengths [14]. The reaction was started by the addition of 1mM (or different concentration) glucose 6 phosphate, and the production of NADPH recorded for 10–15 min at room temperature. In separate sets of experiments, NADPH production was recorded for a similar period of time at 37°C. For comparison purposes, NADPH production was measured in an Agilent 8453 spectrophotometer equipped with data acquisition software according to the procedure of Senesi et al. [15]. Calibration was carried out by adding known amounts of NADPH standards to aliquots of heat-inactivated permeabilized cells under similar experimental conditions.

#### Cortisol production

The activity of the 11-β-hydroxysteroid-dehydrogenase-1 (11-β-≅ HSD1) in reducing cortisone to cortisol was measured as reported elsewhere [14]. Briefly, HepG2 cells in culture were incubated in the presence of 5μM cortisone for 12 hours. The cells were then placed on ice, washed once with PBS, and extracted with 500 μl ice cold methanol. The methanol mixture was stored at −20°C. After thawing, the methanol mixture was centrifuged at 18,000 rpm (20,000 g for 10 min at $ C in a Beckman J20 centrifuge. The supernatants were collected, and their cortisone and cortisol contents measured by HPLC (BioRad 1740) using a 5 μm mash C18 reverse phase column HPLC (Waters), and an isocratic methanol: water, 58:42 (vol/vol) mobile phase at a flow rate of 0.7 ml/min. Absorbance was detected at 245 nm (Biorad 1740 UV detector). The peaks corresponding to cortisone and cortisol and their retention time were determined via standards injection [14].

### Statistical analysis

Data are reported as means ± S.E. of at least four different preparations for each experimental condition, each tested in duplicate. Data were analyzed by Student T- test set at p<0.05 for significance.

## Results

**Figure 1:**
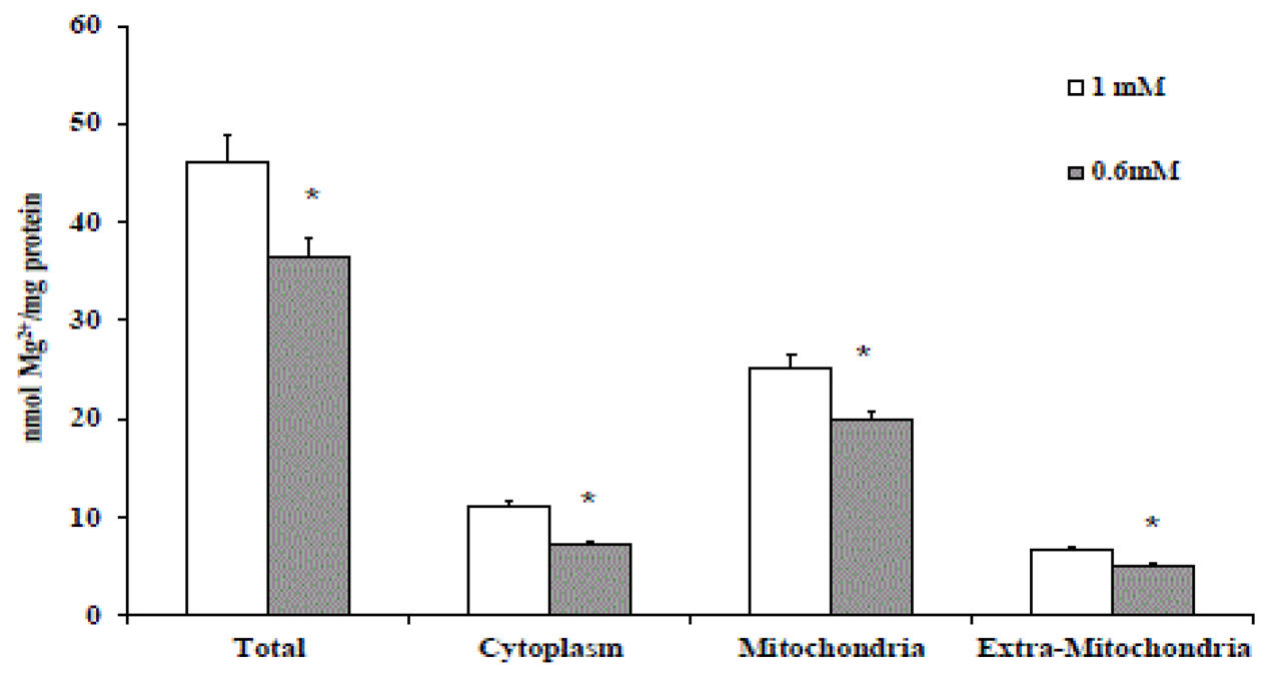
Total cellular Mg^2+^ content and intracellular Mg^2+^ distribution in HepG2 cells grown in the presence of 1 mM or 0.6 mM [Mg^2+^]o.

HepG2cells cultured in the presence of 0.5 mM extracellular Mg^2+^ presented ~20% less total cellular Mg^2+^ than HepG2 cells maintained in the presence of physiological 1 mM [Mg^2+^]o (34.2 ± 0.9 versus 42.3 ± 1.2 nmol Mg^2+^/mg protein, n=8, p<0.05) (Figure1). Assessment of the intracellular Mg^2+^ distribution as reported under Materials and Methods confirmed that Mg^2+^ content decreased in the cytoplasm, mitochondria, and post mitochondrial pools (Figure 1). HepG2 cells maintained in 0.6 mM [Mg^2+^]o also showed a significant 18% decrease in total ATP content (4.35 ± 0.8 vs. 4.18 ± 1.2 nmol/mg protein, n=10, p<0.05).

HepG2 cells maintained in culture in the presence of 1 mM (physiological) or 0.6 mM (Mg^2+^-deficient) extracellular Mg^2+^ were assessed for total cellular Mg^2+^ content and intracellular Mg^2+^ compartmentalization as described under Materials and Methods. Figure 1 onset reports the time points at which Mg^2+^ content was assessed and the various agents added to the cells. The data are means ± S.E. of 4 different preparations, each assessed in duplicate. *Statistical significant versus the corresponding value in 1 mM HepG2 cells.

**Figure 2:**
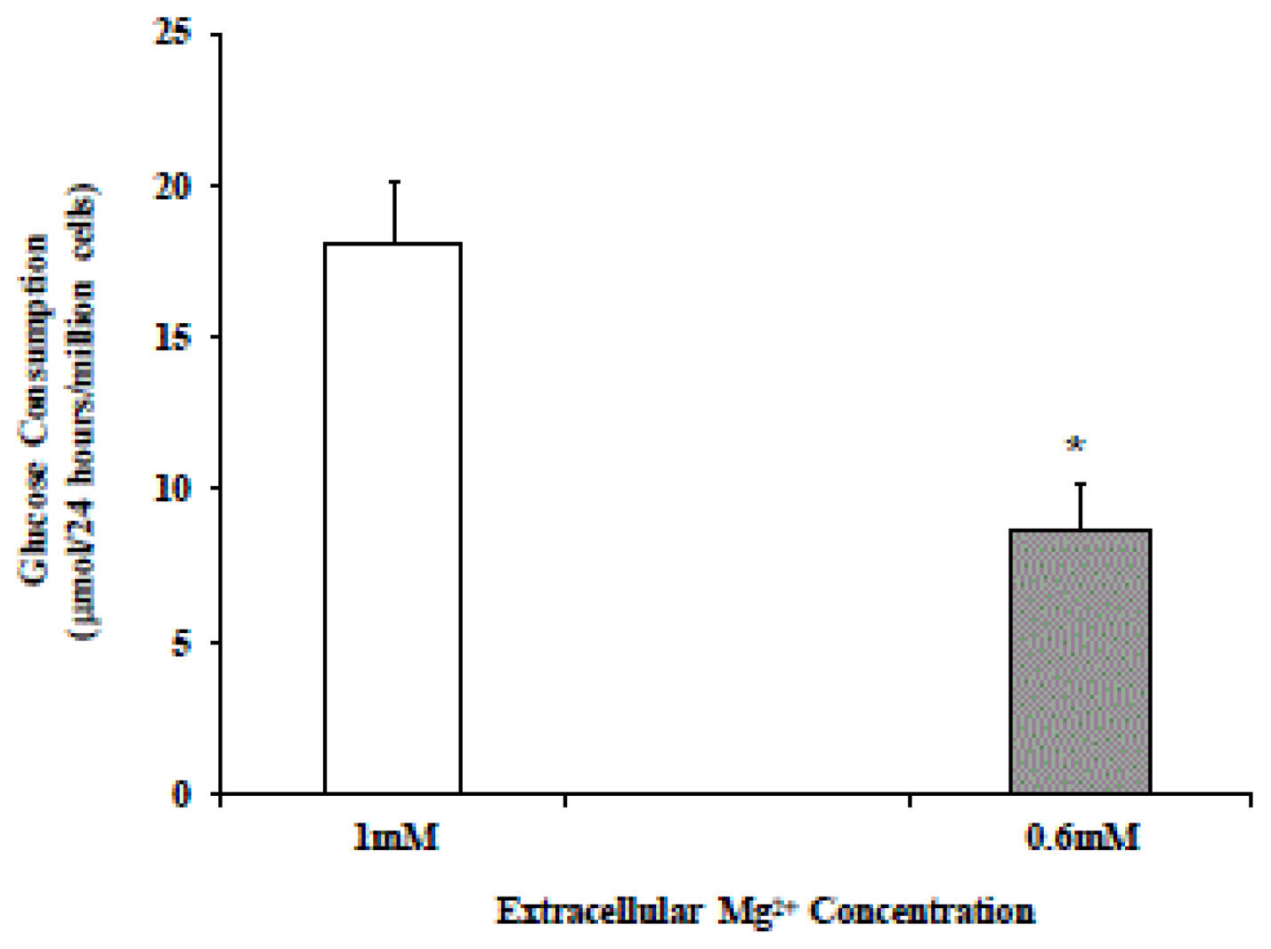

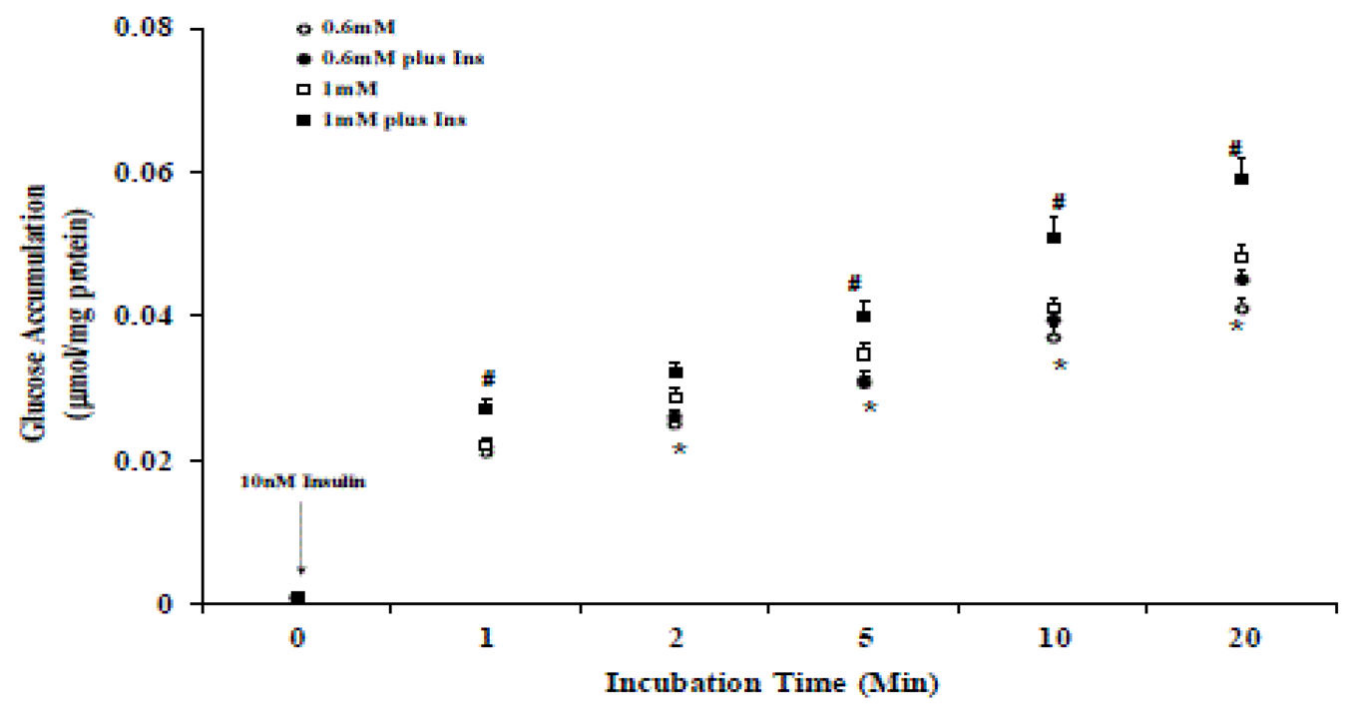
Glucose disappearance from the culture medium (Figure 2A) and glucose accumulation (Figure 2B) in HepG2 cells cultured in the presence of 0.6 mM or 1 mM external Mg^2+^.

Our and others laboratories [13,16] have previously reported that a reduction in extracellular Mg^2+^ concentration impairs glucose accumulation into the cells. To determine whether the reduction in ATP content depended on a reduced transport of glucose into the cells, we measured the changes in medium glucose content at 24 hour intervals by glucose enzymatic kit. Our results indicated that HepG2 cells in 0.6 mM [Mg^2+^]o accumulated approximated 50% less glucose than HepG2 cells in 1 mM [Mg^2+^] (Figure 2A). Because the reduced glucose utilization could depend on changes in the number of cells and their reduplication rate, we measured glucose transport into the cells by 3H-2-deoxy-glucose radio-isotopic distributions, normalized per number of cells. As Figure 2B shows, 0.6 mM HepG2 cells accumulated less glucose (~85%) than 1 mM HepG2 cells under basal conditions. The glucose accumulation rate in 0.6 mM HepG2 cells did not increase upon administration of 10 nM insulin (Figure 2B), whereas it increased significantly in 1 mM HepG2 cells. Similar results were obtained when glucose was labelled with 3H-3-methyl-glucose or 14C-glucose (data not shown), or when glucose concentrations higher than 100 μM were used (data now shown).

HepG2 cells were maintained in culture in the presence of 0.6 mM or 1 mM extracellular Mg^2+^. Glucose disappearance from the medium was measured by glucose assay kit in aliquots of the culture medium withdrawn at 24 hours interval (Figure 2A). Accumulation of glucose (100μM labeled with 0.5mCi/ml 3H-2-deoxy-glucose) into the cells was measured as the amount of radioactivity accumulated within the cells at the indicated time points (Figure 2B). Where indicated, insulin (10 nM) was added to the incubation system, and glucose accumulation carried out for 20 min. Data are means ± S.E. of 4 different experiments, each carried out in duplicate, for all the experimental conditions reported.

*Statistical significant versus the corresponding value in 1 mM HepG2 cells.

#Statistical significant versus the corresponding value in 1 mM HepG2 cells in the absence of insulin stimulation.

**Figure 3:**
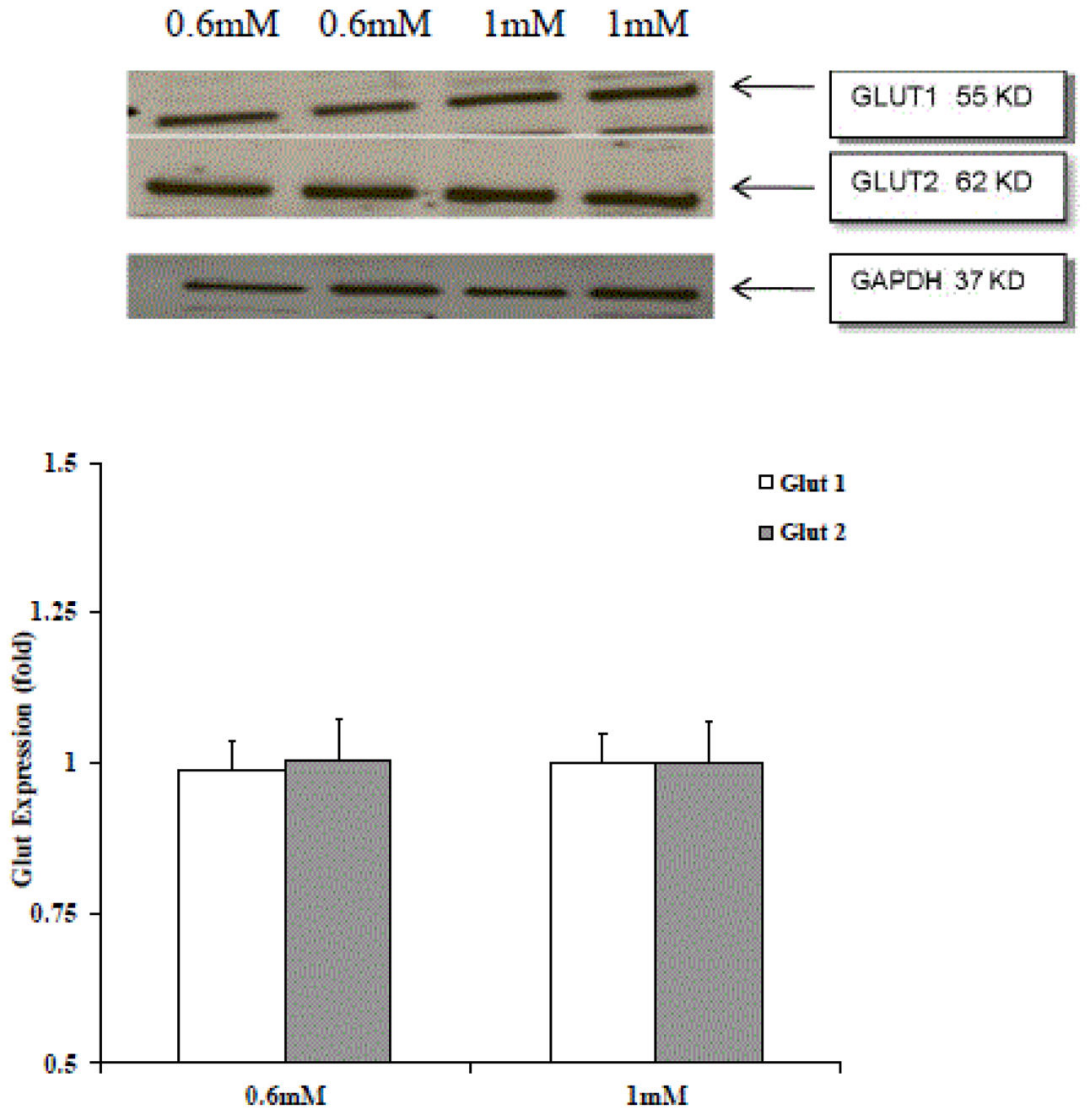
Glut2 and Glut1 expression in HepG2 cells cultured in the presence of 0.6 mM or 1 mM extracellular Mg^2+^

To determine whether the defect in glucose accumulation depended on a different expression of glucose transporters Western blot analysis and mRNA expression were carried out for Glut2 and Glut1 under our experimental conditions. As Figure 3 shows, no significant differences in Glut 2 and Glut 1 protein expression were observed.

Assessment of Glut2 and Glut1 protein expression in HepG2 cells was carried by Western blot analysis using commercially available antibodies against the glucose transporters. Densitometry was carried out using Scion program (NIH) as indicated under Materials and Methods. Data are means ± S.E. of three different preparations, each tested in duplicate.

**Figure 4:**
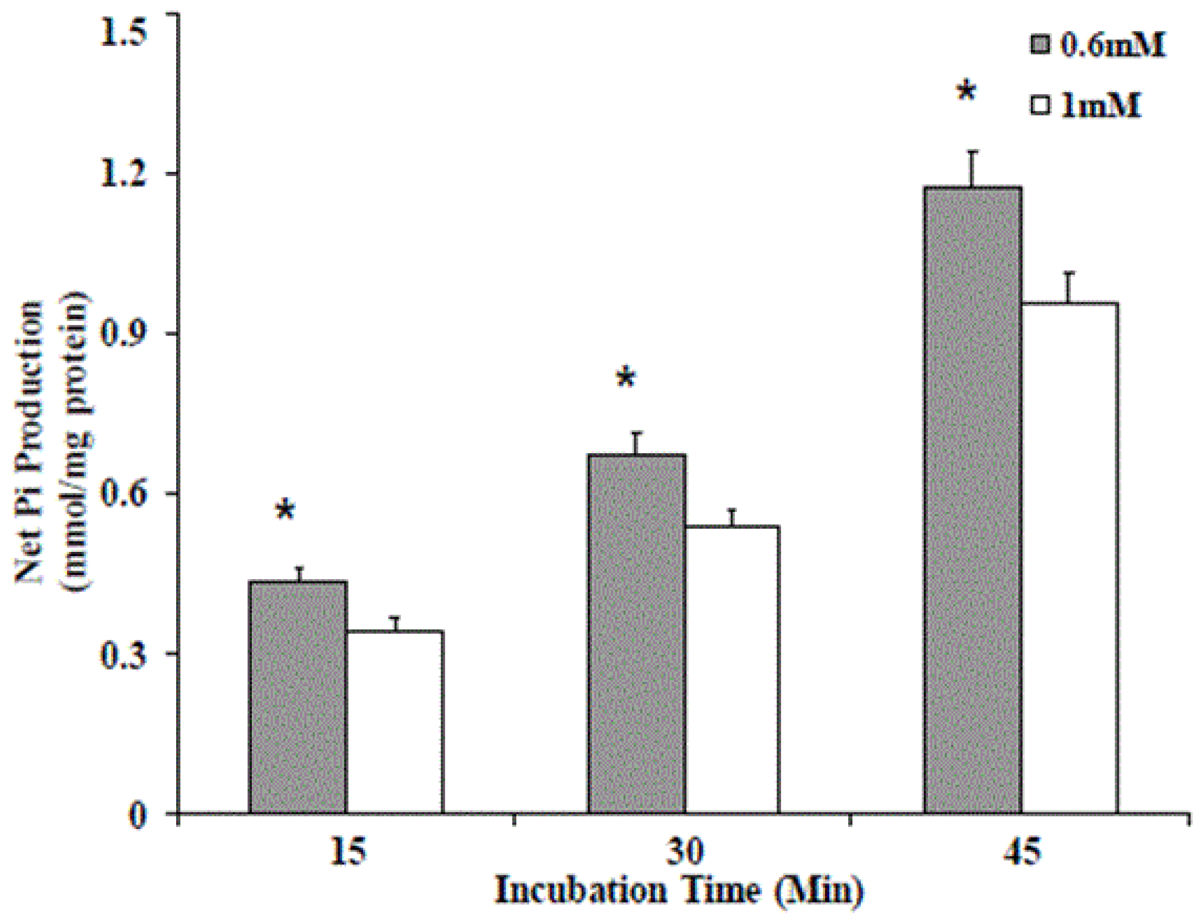
Glucose 6-phosphate hydrolysis in HepG2 cells

We have previously reported that a decrease in cytoplasmic Mg^2+^ content enhances the transport of glucose 6-phosphate (G6P) into the hepatic endoplasmic reticulum and its hydrolysis by the glucose 6-phosphatase. These results were obtained in both freshly isolated hepatocytes [10] and in microsomal vesicles from livers of Mg^2+^ deficient animals [17]. A similar increase in G6P transport and hydrolysis was observed in permeabilized HepG2 cells (Figure 4), confirming our previous experimental results.

In addition to being hydrolyzed by the G6Pase, G6P is also oxidized within the hepatic ER to 6-phosphogluconolactone by the glucose 6-phosphate dehydrogenase in a process associated with the production of endo-luminal NADPH [14]. The reaction catalyzed by the G6PD represents the limiting step in the pentose shunt, an alternative energetic pathway within the hepatocyte.

HepG2 cells were maintained in culture in the presence of 0.6 mM or 1 mM extracellular Mg^2+^. At confluence, cells were harvested, and permeabilized by digitonin addition (50 μg/ml). The cells were then washed and incubated in a cytosol-like medium in the presence of 1 mM G6P, at 37°C. Hydrolysis of G6P by glucose 6-phosphatase was measured as the amount of Pi released into the incubation medium at specific time points. Data are means ± S.E. of 5 different experiments, each carried out in duplicate, for all the experimental conditions reported.

*Statistical significant versus the corresponding value in 1 mM HepG2 cells.

**Figure 5:**
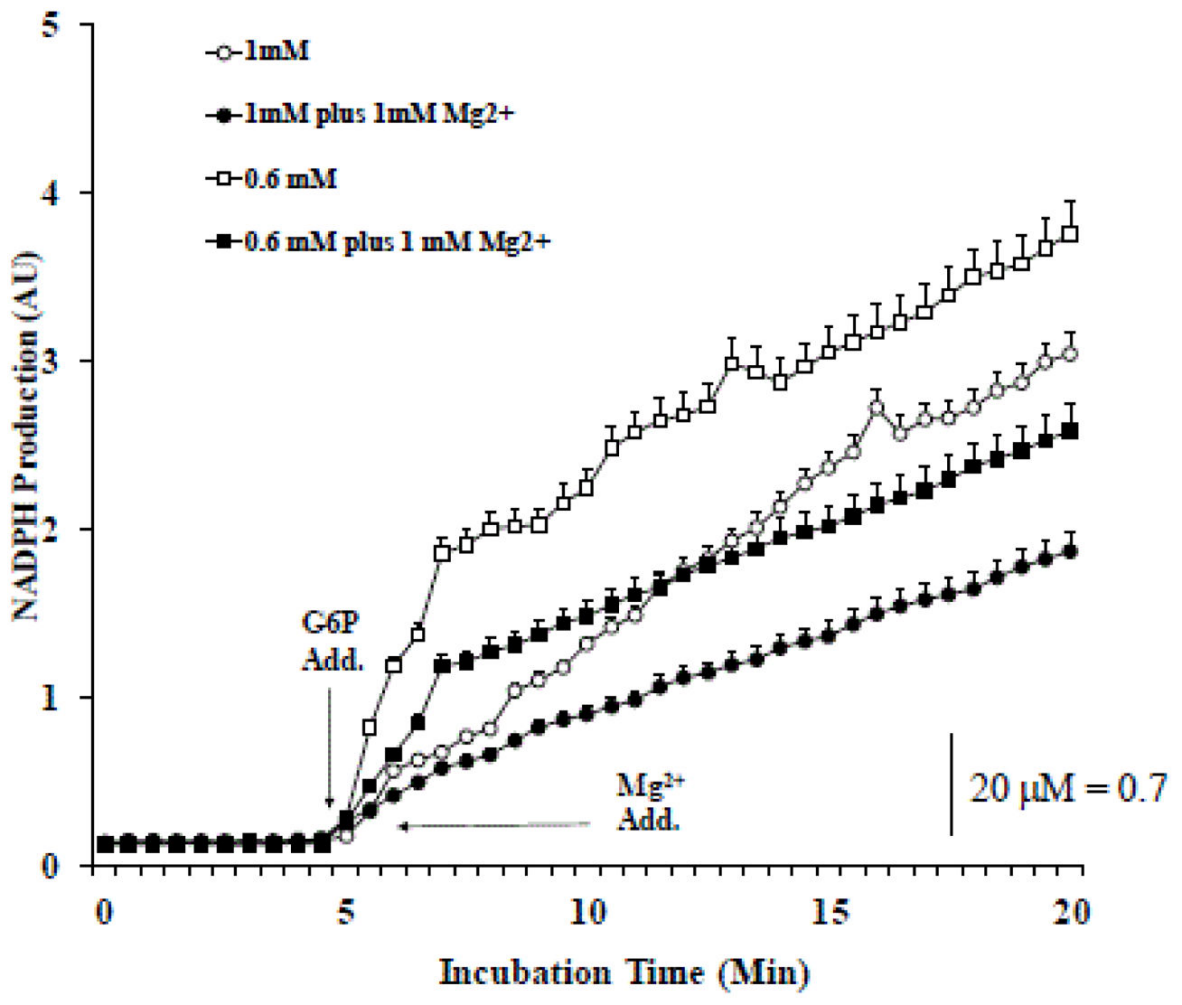

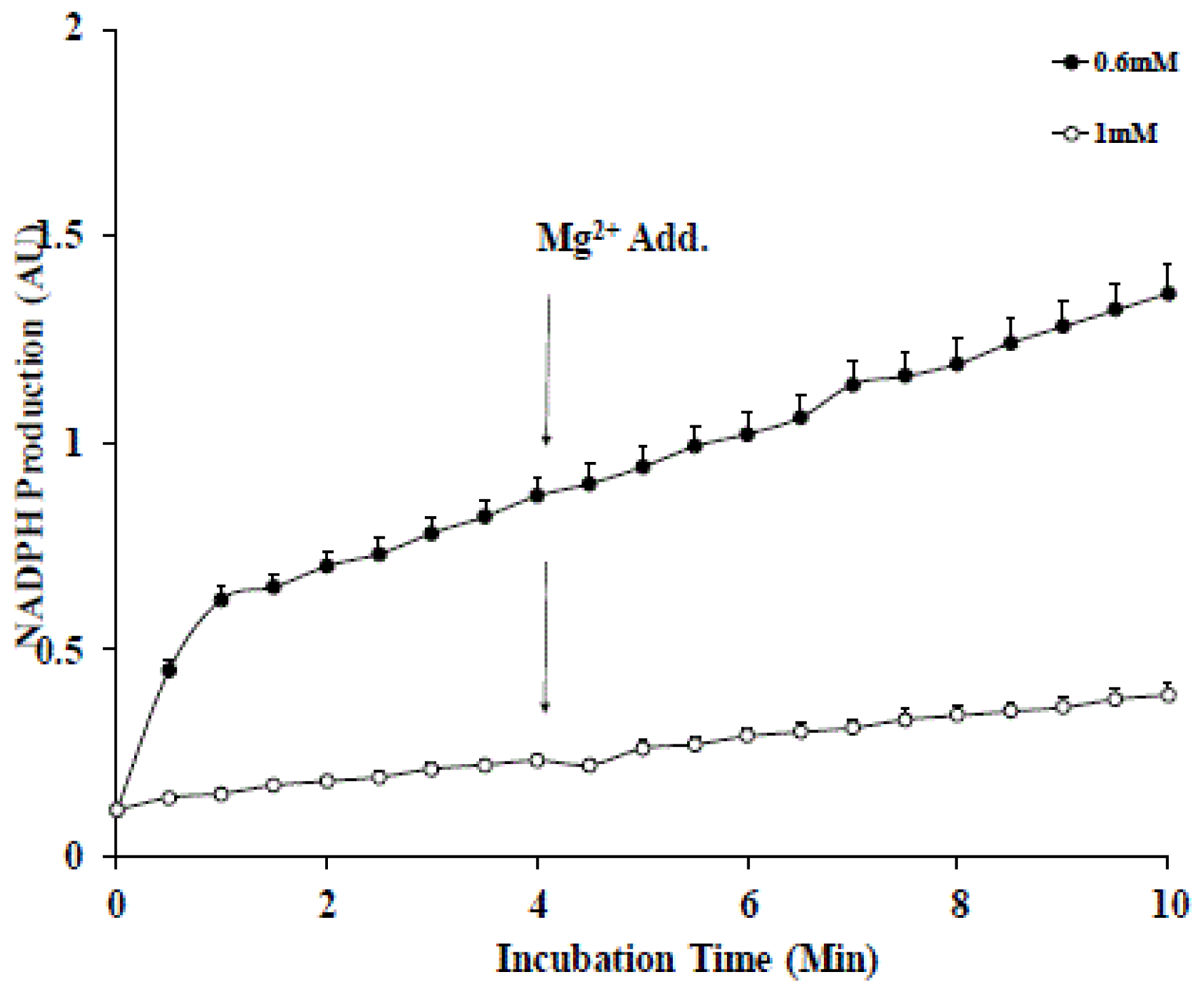
**Figure 5A.** 0.6 mM HepG2 cells produced twice the amount of NADPH than 1 mM HepG2 cells **Figure 5.** Glucose 6-phosphate dehydrogenase activity in HepG2 cells

To determine whether also this alternative pathway was enhanced in Mg^2+^ deficient cells, the production of NADPH was measured in HepG2 cells in the presence of various amounts of exogenous G6P. The results reported in Figure 5A indicate 0.6 mM HepG2 cells produced almost twice the amount of NADPH than 1 mM HepG2 cells. The NADPH production rate in 0.6 mM HepG2 cells was reduced by the addition of excess Mg^2+^ excess prior to G6P administration (Figure 5A) but not when a similar (Figure 5B) or a higher (not shown) dose of Mg^2+^ was added after the G6P administration and the NADPH production was already near maximal.

HepG2 cells were maintained in culture in the presence of 0.6 mM or 1 mM extracellular Mg^2+^. At confluence, cells were harvested, and permeabilized by digitonin addition (50μg/ml). The cells were then washed and incubated in a cytosol-like medium in the presence of 1 mM G6P, at 37°C. NADPH as the by-product of G6P conversion to 6-phosphogluconolactone by the reticular G6PD was measured fluorimetrically as described under Materials and Methods. Calibration was carried out as described in the Methods section. The inhibitory effect of exogenous 1 mM Mg^2+^ added prior to G6P addition is reported in Figure 5A. The lack on an inhibitory effect of exogenous 1 mM Mg^2+^ added following G6P addition is reported in Figure 5B. Data are means ± S.E. of 3 different experiments for all the experimental conditions reported. In both Figure 5A and Figure 5B, all the data points for 0.6 mM following G6P addition and Mg^2+^ addition are statistical significant versus the corresponding values in 1 mM HepG2 cells. Labeling is omitted for simplicity.

Experimental evidence indicates that the production of luminal NADPH through G6PD is coupled to the conversion of cortisone to cortisol by the 11-β-hydroxysteroid-dehydrogenase-1 [14]. Renewed attention has been paid to this enzyme as an increase in its activity and/or expression and the associated production of cortisol have been considered one of the possible causes for increased insulin resistance in certain forms of type-2 diabetes.

**Figure 6:**
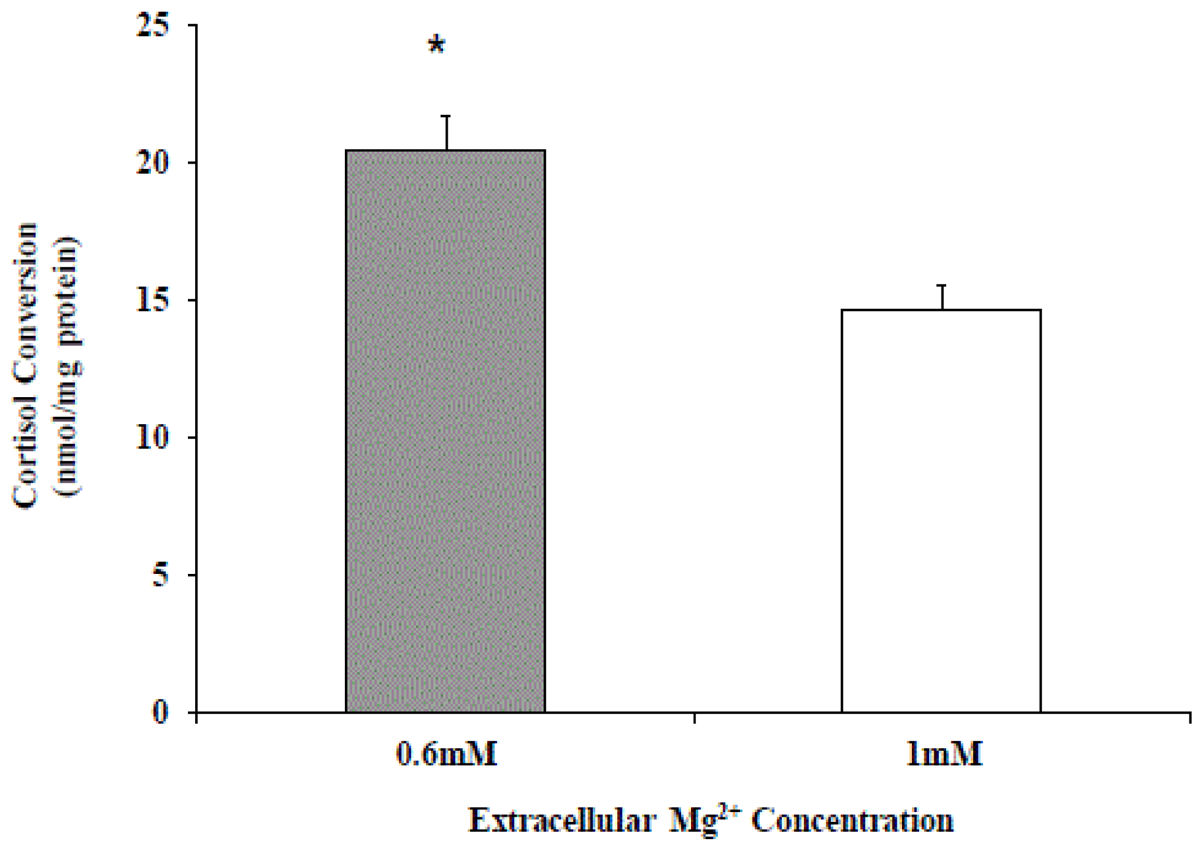
Cortisol production in HepG2 cells

To establish whether Mg^2+^-deficient HepG2 cells could indeed convert cortisone to cortisol at an higher rate than HepG2 cells maintained in the presence of physiological extracellular Mg^2+^, exogenous cortisone was administered for 12 hours to cells in culture, and the cortisone to cortisol conversion measured by HPLC. The results reported in Figure 6 indicate that Mg^2+^-deficient HepG2 cells produced approximately 30% more cortisol than cells maintained in physiological Mg^2+^.

HepG2 cells were maintained in culture in the presence of 0.6 mM or 1 mM extracellular Mg^2+^. Cortisone (5 μM) was added for 12 hours to cells in culture. The cells were then harvested and processed as described in the Methods section. Cortisone to cortisol conversion by the 11-b-OHSD1 was assessed by HPLC and normalized per mg of protein. Data are means ± S.E. of 3 different experiments for all the experimental conditions reported. *Statistical significant versus the corresponding value in 1 mM HepG2 cells.

## Discussion

The incidence of metabolic syndrome in Western countries has markedly increased in the last two decades. Altered ion homeostasis including hypomagnesaemia and reduced cellular Mg^2+^ levels have frequently been observed in patients affected by metabolic syndrome, raising the question as to whether the Mg^2+^ deficiency is consequence or cause of the pathology onset.

Although carried out in liver cells in culture, the results reported here suggest that Mg^2+^ deficiency is a contributing factor. Induction of Mg^2+^ deficiency in liver cells impairs glucose metabolism to a significant extent and in different manners. For one, the decrease in extracellular and cellular Mg^2+^ content reduces the ability of these hepatocytes to accumulate and utilize glucose properly, resulting in a decrease in ATP content. This decrease in glucose accumulation and utilization does not depend on changes in glucose transport expression as both Glut2, the predominant glucose transporter in these cells, and Glut1 - which is also present to an extent in the cells - are expressed to a comparable extent in cells grown in the presence of low (0.6 mM) or physiological (1 mM) extracellular Mg^2+^. As we have not measured directly the glucokinase activity, we cannot exclude the possibility that the reduced glucose entry observed in Mg^2+^ deficient cells depends on a reduced activity of this enzyme. However, the reported data on increased G6P transport and hydrolysis by the endo-reticular glucose 6-phosphatase suggest, albeit indirectly, that G6P generated by the glucokinase is routed more rapidly towards the endoplasmic reticulum, resulting in an enhanced glucose-to G6P-to glucose futile cycle [18], with possible implications for the net ATP loss detected in these cells.

Perhaps more pertinent to the metabolic syndrome onset are our results on an increased activity of the glucose 6-phosphate dehydrogenase, the other reticular enzymes involved in metabolizing G6P. This enzyme represents the limiting step in the pentose phosphate shunt pathway, and the conversion of G6P to 6-phosphogluconolactone catalyzed by the enzyme is coupled to the reduction of NADP to NADPH. This endo-reticular pool of reduced pyridine nucleotide can then be used for fatty acids and cholesterol synthesis within the hepatocyte. More importantly, the NADPH reticular pool can be used by the 11-β-hydrosteroid dehydrogenase-1 to reduce cortisone to cortisol within the hepatocyte as attested by the 30% increase in cortisol production under our experimental conditions. Recently, experimental and clinical evidence has associated the increased expression and/or activity of this enzyme with insulin resistance and obesity [14], two stigmata of the metabolic syndrome [3,4]. Preliminary experiments in our laboratory support the idea that Mg^2+^ deficiency results in reduced insulin receptor responsiveness (Romani, personal observation). It remains to be elucidated as to whether the decrease in insulin response depends directly on the Mg^2+^ deficiency, which impairs the autophosphorylation of the insulin receptor and the subsequent activation of the insulin receptor substrate, as observed in skeletal muscles of Mg^2+^ deficient animals [19], or indirectly through the G6PDNADPHcortisol up-regulation observed under our experimental conditions. In this respect, studies are being carried out in our laboratory to determine whether Mg^2+^ deficiency also promotes G6PD gene expression in addition to increasing G6PD enzymatic activity.

Because the hepatocytes are able to interchangeably utilize glucose or fatty acids for energetic purposes, it could be argued that the increased G6PD activity should results in more NADPH being routed towards the synthesis of fatty acids, which the hepatocytes should be able to utilize as energetic substrates for metabolic purposes without experiencing a decrease in ATP content. Yet, this does not appear to be the case. In addition, both fetal and bovine calf serum, which are commonly utilized in cell culture to support cell growth, have significant levels of short chain fatty acids that the HepG2 cells could use for energy purposes. Thus, the possibility that the reduced ATP content measured in HepG2 Mg^2+^-deficient cells might depend on energetic substrate restriction (i.e. only glucose in the culture medium) is also not supported. Although more detailed experiments need to be carried out to elucidate the reasons behind the inability of Mg^2+^ deficient HepG2 cells to sustain their ATP level, it is possible that less than optimal Mg^2+^ content within the mitochondria, another major cellular pool for this cation [6] affects the organelle β-oxidation.

Irrespective of the mechanism behind the reduced ATP production (or increased ATP consumption), an increase in G6PD activity has been related to increased lipid dysfunction and insulin resistance [20], thus landing support to our observation and its experimental and clinical relevance.

To our knowledge, this is the first study that has provided a cause-effect link between Mg^2+^ deficiency and some of the metabolic alterations present in the metabolic syndrome, namely altered glucose metabolism, insulin resistance, and possibly obesity. Because of the novelty of our observation, several questions are still in need of a more thoroughly investigation including the validation of our data in human subjects.
